# 3-(4-Bromo­phenyl­sulfin­yl)-2,5,6-trimethyl-1-benzo­furan

**DOI:** 10.1107/S1600536813017753

**Published:** 2013-07-06

**Authors:** Hong Dae Choi, Pil Ja Seo, Uk Lee

**Affiliations:** a Department of Chemistry, Dongeui University, San 24 Kaya-dong, Busanjin-gu, Busan 614-714, Republic of Korea; b Department of Chemistry, Pukyong National University, 599-1 Daeyeon 3-dong, Nam-gu, Busan 608-737, Republic of Korea

## Abstract

In the title compound, C_17_H_15_BrO_2_S, the dihedral angle between the mean plane [r.m.s. deviation = 0.003 (2) Å] of the benzo­furan ring system and the mean plane [r.m.s. deviation = 0.006 (2) Å] of the 4-bromo­phenyl ring is 83.09 (7)°. In the crystal, weak C—H⋯π inter­actions are observed.

## Related literature

For background information and the crystal structures of related compounds, see: Choi *et al.* (2010*a* ▶,*b* ▶, 2012 ▶).
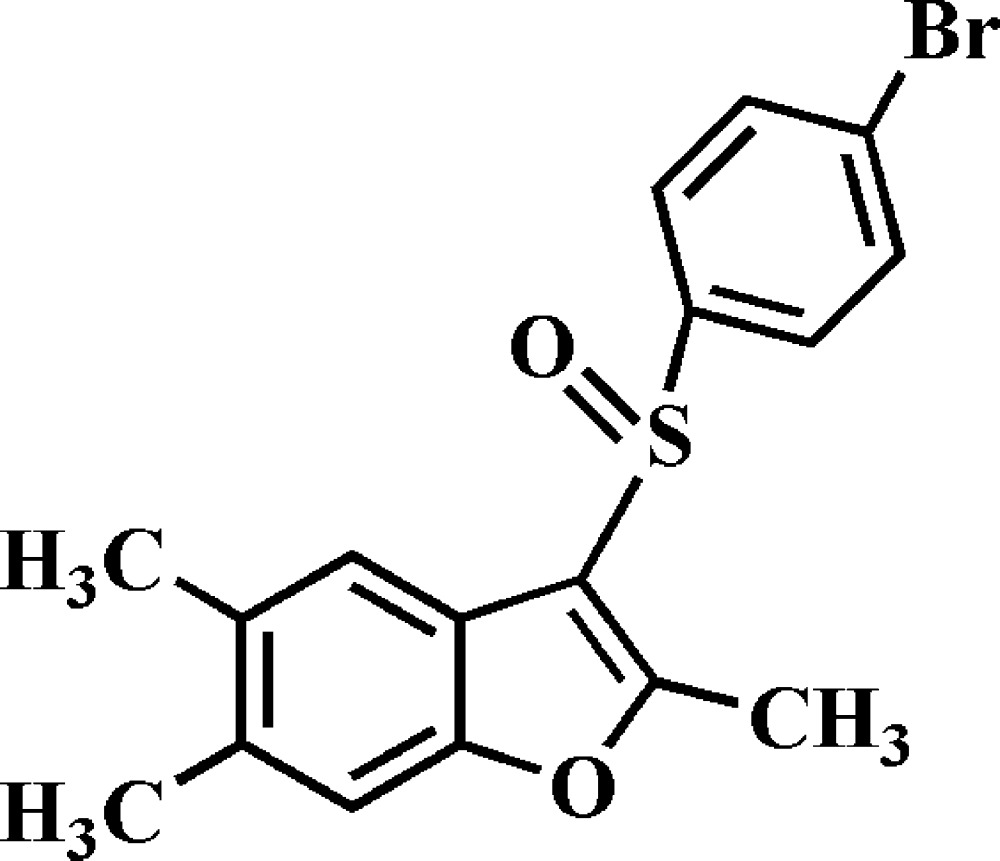


## Experimental

### 

#### Crystal data

C_17_H_15_BrO_2_S
*M* *_r_* = 363.26
Monoclinic, 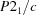

*a* = 20.0084 (8) Å
*b* = 7.1890 (3) Å
*c* = 10.7804 (4) Å
β = 101.478 (2)°
*V* = 1519.65 (10) Å^3^
*Z* = 4
Mo *K*α radiation
μ = 2.84 mm^−1^
*T* = 173 K
0.37 × 0.26 × 0.05 mm

#### Data collection

Bruker SMART APEXII CCD diffractometer
Absorption correction: multi-scan (*SADABS*; Bruker, 2009 ▶) *T*_min_ = 0.425, *T*_max_ = 0.746
26485 measured reflections
3791 independent reflections
2928 reflections with *I* > 2σ(*I*)
*R*_int_ = 0.071

#### Refinement

*R*[*F* ^2^ > 2σ(*F* ^2^)] = 0.041
*wR*(*F* ^2^) = 0.114
*S* = 1.05
3791 reflections
193 parameters
H-atom parameters constrained
Δρ_max_ = 0.75 e Å^−3^
Δρ_min_ = −0.86 e Å^−3^

### 

Data collection: *APEX2* (Bruker, 2009 ▶); cell refinement: *SAINT* (Bruker, 2009 ▶); data reduction: *SAINT*; program(s) used to solve structure: *SHELXS97* (Sheldrick, 2008 ▶); program(s) used to refine structure: *SHELXL97* (Sheldrick, 2008 ▶); molecular graphics: *ORTEP-3 for Windows* (Farrugia, 2012 ▶) and *DIAMOND* (Brandenburg, 1998 ▶); software used to prepare material for publication: *SHELXL97*.

## Supplementary Material

Crystal structure: contains datablock(s) global, I. DOI: 10.1107/S1600536813017753/nc2313sup1.cif

Structure factors: contains datablock(s) I. DOI: 10.1107/S1600536813017753/nc2313Isup2.hkl

Supplementary material file. DOI: 10.1107/S1600536813017753/nc2313Isup3.cml

Additional supplementary materials:  crystallographic information; 3D view; checkCIF report

## Figures and Tables

**Table 1:** Hydrogen-bond geometry (Å, °) *Cg*1 and *Cg*2 are the centroids of the C1/C2/C7/O1/C8 furan ring and the C2–C7 benzene ring, respectively.

*D*—H⋯*A*	*D*—H	H⋯*A*	*D*⋯*A*	*D*—H⋯*A*
C9—H9*C*⋯*Cg*1^i^	0.98	2.89	3.537 (4)	124
C11—H11*C*⋯*Cg*2^ii^	0.98	2.78	3.714 (4)	159

Symmetry codes: (i) 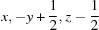; (ii) 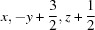.

**Fig. 1.**
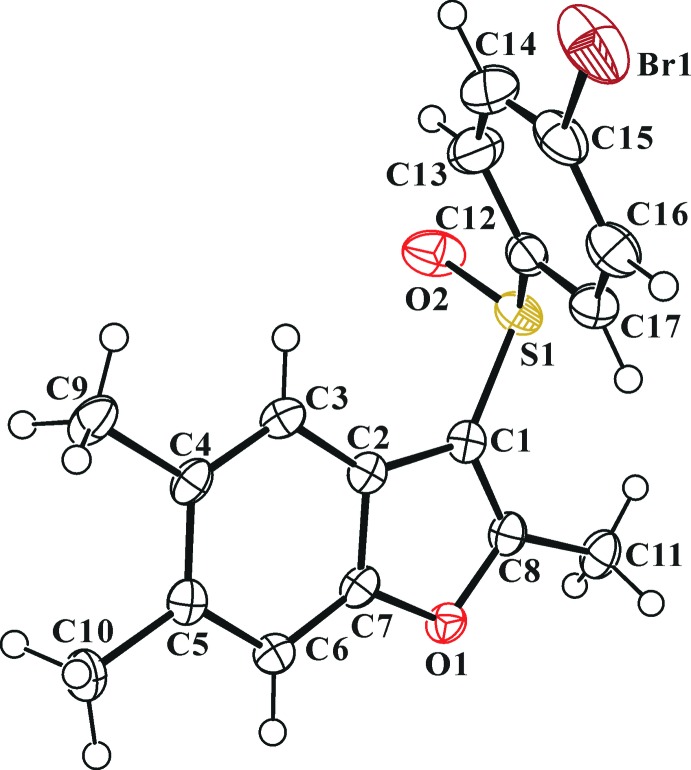
The molecular structure of the title compound with the atom numbering scheme. Displacement ellipsoids are drawn at the 50% probability level. H atoms are presented as small spheres of arbitrary radius.

**Fig. 2.**
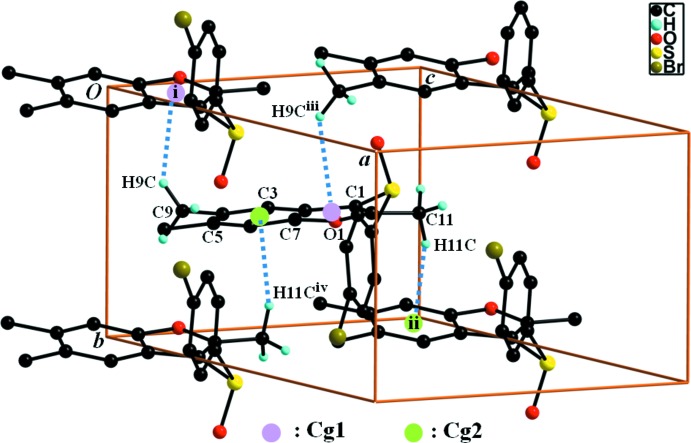
Fig. 2. A view of the C—H···π interactions (dotted lines) in the crystal structure of the title compound. H atoms that does not participate in this interaction are omitted for clarity.Symmetry codes: (i) x, - y + 1/2, z - 1/2; (ii) x, - y + 3/2, z + 1/2; (iii) x, - y +1/2, z + 1/2; (iv) x, - y + 3/2, z - 1/2.]

## supplementary crystallographic information

### Comment

As a part of our continuing study of 2,5-dimethyl-1-benzofuran derivatives containing 4-fluorophenylsulfinyl (Choi *et al.*, 2010*a*), 4-chlorophenylsulfinyl (Choi *et al.*, 2010*b*) and 4-bromophenylsulfinyl (Choi *et al.*, 2012) substituents in 3-position, we report here the crystal structure of the title compound.

In the title molecule the benzofuran unit is essentially planar, with a mean deviation of 0.003 (2) Å from the least-squares plane defined by the nine constituent atoms (Fig. 1). The 4-bromophenyl ring is essentially planar, with a mean deviation of 0.006 (2) Å from the least-squares plane defined by the six constituent atoms. The dihedral angle between the mean plane of the benzofuran ring system and the 4-bromophenyl ring is 83.09 (7)°. In the crystal structure the molecules are connected by weak C—H···π interactions (Table 1 and Fig. 2), Cg1 and Cg2 are the centroids of the C1/C2/C7/O1/C8 furan ring and the C2–C7 benzene ring, respectively)

### Experimental

3-Chloroperoxybenzoic acid (77%, 202 mg, 0.9 mmol) was added in small portions to a stirred solution of 3-(4-bromophenylsulfanyl)-2,5,6-trimethyl-1-benzofuran (278 mg, 0.8 mmol) in dichloromethane (30 mL) at 273 K. After being stirred at room temperature for 4h, the mixture was washed with saturated sodium bicarbonate solution and the organic layer was separated, dried over magnesium sulfate, filtered and concentrated at reduced pressure. The residue was purified by column chromatography (hexane–ethyl acetate, 4:1 v/v) to afford the title compound as a colorless solid [yield 71%, m.p. 446–447 K; *R*_f_ = 0.41 (hexane-ethyl acetate, 4:1 v/v)]. Single crystals suitable for X-ray diffraction were prepared by slow evaporation of the solvent from a solution of the title compound in ethyl acetate at room temperature.

### Refinement

All H atoms were positioned geometrically (methyl H atoms allowed to rotate but not to tip) and refined with *U*_iso_(H) = 1.2*U*_eq_(C) (1.5 for methyl H atoms) using a riding model with C—H = 0.95 Å for aryl and 0.98Å for methyl H atoms.

### Crystal data

**Table:**

C_17_H_15_BrO_2_S	*F*(000) = 736
*M**_r_* = 363.26	*D*_x_ = 1.588 Mg m^−^^3^
Monoclinic, *P*2_1_/*c*	Melting point = 446–447 K
Hall symbol: -P 2ybc	Mo *K*α radiation, λ = 0.71073 Å
*a* = 20.0084 (8) Å	Cell parameters from 6275 reflections
*b* = 7.1890 (3) Å	θ = 3.1–27.2°
*c* = 10.7804 (4) Å	µ = 2.84 mm^−^^1^
β = 101.478 (2)°	*T* = 173 K
*V* = 1519.65 (10) Å^3^	Block, colourless
*Z* = 4	0.37 × 0.26 × 0.05 mm

### Data collection

**Table:**

Bruker SMART APEXII CCD diffractometer	3791 independent reflections
Radiation source: rotating anode	2928 reflections with *I* > 2σ(*I*)
Graphite multilayer monochromator	*R*_int_ = 0.071
Detector resolution: 10.0 pixels mm^-1^	θ_max_ = 28.4°, θ_min_ = 2.1°
φ and ω scans	*h* = −26→26
Absorption correction: multi-scan (*SADABS*; Bruker, 2009)	*k* = −7→9
*T*_min_ = 0.425, *T*_max_ = 0.746	*l* = −14→14
26485 measured reflections	

### Refinement

**Table:**

Refinement on *F*^2^	Primary atom site location: structure-invariant direct methods
Least-squares matrix: full	Secondary atom site location: difference Fourier map
*R*[*F*^2^ > 2σ(*F*^2^)] = 0.041	Hydrogen site location: difference Fourier map
*wR*(*F*^2^) = 0.114	H-atom parameters constrained
*S* = 1.05	*w* = 1/[σ^2^(*F*_o_^2^) + (0.0459*P*)^2^ + 0.8553*P*] where *P* = (*F*_o_^2^ + 2*F*_c_^2^)/3
3791 reflections	(Δ/σ)_max_ = 0.001
193 parameters	Δρ_max_ = 0.75 e Å^−^^3^
0 restraints	Δρ_min_ = −0.86 e Å^−^^3^

### Special details

**Table:**

Geometry. All esds (except the esd in the dihedral angle between two l.s. planes) are estimated using the full covariance matrix. The cell esds are taken into account individually in the estimation of esds in distances, angles and torsion angles; correlations between esds in cell parameters are only used when they are defined by crystal symmetry. An approximate (isotropic) treatment of cell esds is used for estimating esds involving l.s. planes.
Refinement. Refinement of F^2^ against ALL reflections. The weighted R-factor wR and goodness of fit S are based on F^2^, conventional R-factors R are based on F, with F set to zero for negative F^2^. The threshold expression of F^2^ > 2sigma(F^2^) is used only for calculating R-factors(gt) etc. and is not relevant to the choice of reflections for refinement. R-factors based on F^2^ are statistically about twice as large as those based on F, and R- factors based on ALL data will be even larger.

### Fractional atomic coordinates and isotropic or equivalent isotropic displacement parameters (Å^2^)

**Table:**

	*x*	*y*	*z*	*U*_iso_*/*U*_eq_	
Br1	0.471187 (16)	0.90385 (6)	0.33352 (3)	0.06164 (16)	
S1	0.30484 (3)	0.38470 (9)	0.64879 (6)	0.02963 (16)	
O1	0.11729 (8)	0.5558 (2)	0.62907 (13)	0.0245 (4)	
O2	0.30702 (10)	0.1917 (3)	0.5990 (2)	0.0456 (5)	
C1	0.22159 (11)	0.4684 (3)	0.60389 (19)	0.0230 (5)	
C2	0.17882 (11)	0.4857 (3)	0.47877 (19)	0.0218 (4)	
C3	0.18654 (12)	0.4633 (3)	0.35339 (19)	0.0240 (5)	
H3	0.2292	0.4267	0.3354	0.029*	
C4	0.13130 (12)	0.4951 (3)	0.25574 (19)	0.0242 (5)	
C5	0.06756 (12)	0.5501 (3)	0.28199 (19)	0.0230 (4)	
C6	0.06000 (11)	0.5739 (3)	0.40622 (19)	0.0230 (5)	
H6	0.0178	0.6124	0.4253	0.028*	
C7	0.11582 (11)	0.5398 (3)	0.50083 (18)	0.0220 (4)	
C8	0.18216 (12)	0.5111 (3)	0.68885 (19)	0.0245 (5)	
C9	0.13935 (14)	0.4700 (4)	0.1205 (2)	0.0327 (6)	
H9A	0.1875	0.4477	0.1187	0.049*	
H9B	0.1238	0.5826	0.0720	0.049*	
H9C	0.1120	0.3635	0.0831	0.049*	
C10	0.00712 (13)	0.5824 (4)	0.1766 (2)	0.0302 (5)	
H10A	−0.0325	0.6167	0.2123	0.045*	
H10B	−0.0030	0.4683	0.1265	0.045*	
H10C	0.0174	0.6831	0.1221	0.045*	
C11	0.19543 (14)	0.5183 (4)	0.82940 (19)	0.0313 (5)	
H11A	0.2425	0.4795	0.8633	0.047*	
H11B	0.1639	0.4344	0.8607	0.047*	
H11C	0.1887	0.6456	0.8569	0.047*	
C12	0.34419 (11)	0.5327 (4)	0.5498 (2)	0.0270 (5)	
C13	0.38253 (14)	0.4501 (4)	0.4718 (3)	0.0407 (7)	
H13	0.3838	0.3186	0.4644	0.049*	
C14	0.41911 (14)	0.5619 (5)	0.4046 (3)	0.0469 (8)	
H14	0.4451	0.5078	0.3492	0.056*	
C15	0.41750 (12)	0.7519 (5)	0.4186 (2)	0.0379 (7)	
C16	0.37837 (13)	0.8355 (4)	0.4953 (2)	0.0354 (6)	
H16	0.3768	0.9671	0.5022	0.042*	
C17	0.34162 (12)	0.7238 (4)	0.5615 (2)	0.0302 (5)	
H17	0.3146	0.7782	0.6151	0.036*	

### Atomic displacement parameters (Å^2^)

**Table:**

	*U*^11^	*U*^22^	*U*^33^	*U*^12^	*U*^13^	*U*^23^
Br1	0.04327 (19)	0.1047 (4)	0.03699 (18)	−0.03020 (17)	0.00810 (13)	0.01789 (15)
S1	0.0254 (3)	0.0305 (4)	0.0322 (3)	0.0010 (2)	0.0038 (2)	0.0074 (2)
O1	0.0278 (8)	0.0287 (10)	0.0182 (7)	0.0021 (7)	0.0074 (6)	0.0007 (6)
O2	0.0385 (10)	0.0257 (12)	0.0747 (14)	0.0058 (9)	0.0161 (9)	0.0068 (9)
C1	0.0266 (11)	0.0214 (13)	0.0213 (10)	−0.0025 (9)	0.0052 (8)	0.0014 (8)
C2	0.0257 (10)	0.0181 (12)	0.0222 (10)	−0.0018 (9)	0.0059 (8)	0.0016 (8)
C3	0.0291 (11)	0.0215 (13)	0.0231 (10)	0.0034 (10)	0.0094 (8)	0.0024 (8)
C4	0.0351 (12)	0.0189 (12)	0.0199 (9)	0.0002 (10)	0.0088 (8)	0.0002 (8)
C5	0.0286 (11)	0.0179 (12)	0.0220 (10)	−0.0026 (9)	0.0039 (8)	0.0016 (7)
C6	0.0242 (10)	0.0223 (13)	0.0234 (10)	−0.0005 (9)	0.0067 (8)	0.0009 (8)
C7	0.0294 (11)	0.0198 (13)	0.0188 (9)	−0.0017 (9)	0.0094 (8)	0.0007 (7)
C8	0.0299 (11)	0.0223 (13)	0.0208 (10)	−0.0014 (10)	0.0042 (8)	0.0004 (8)
C9	0.0476 (14)	0.0308 (15)	0.0208 (10)	0.0117 (12)	0.0095 (10)	0.0008 (9)
C10	0.0330 (12)	0.0322 (15)	0.0234 (11)	−0.0006 (11)	0.0006 (9)	0.0014 (9)
C11	0.0427 (14)	0.0321 (15)	0.0186 (10)	0.0015 (12)	0.0052 (9)	−0.0006 (8)
C12	0.0225 (10)	0.0335 (15)	0.0242 (10)	−0.0014 (10)	0.0030 (8)	−0.0001 (9)
C13	0.0395 (15)	0.0385 (18)	0.0480 (15)	0.0000 (13)	0.0185 (12)	−0.0072 (12)
C14	0.0393 (16)	0.063 (2)	0.0438 (16)	−0.0047 (14)	0.0222 (13)	−0.0116 (13)
C15	0.0257 (12)	0.059 (2)	0.0288 (12)	−0.0102 (12)	0.0057 (9)	0.0066 (11)
C16	0.0296 (12)	0.0378 (16)	0.0376 (13)	−0.0048 (12)	0.0040 (10)	0.0070 (11)
C17	0.0272 (11)	0.0325 (15)	0.0322 (11)	−0.0010 (10)	0.0086 (9)	0.0005 (9)

### Geometric parameters (Å, º)

**Table:**

Br1—C15	1.892 (3)	C9—H9A	0.9800
S1—O2	1.492 (2)	C9—H9B	0.9800
S1—C1	1.746 (2)	C9—H9C	0.9800
S1—C12	1.796 (2)	C10—H10A	0.9800
O1—C8	1.368 (3)	C10—H10B	0.9800
O1—C7	1.382 (2)	C10—H10C	0.9800
C1—C8	1.358 (3)	C11—H11A	0.9800
C1—C2	1.452 (3)	C11—H11B	0.9800
C2—C7	1.384 (3)	C11—H11C	0.9800
C2—C3	1.400 (3)	C12—C13	1.380 (3)
C3—C4	1.385 (3)	C12—C17	1.382 (4)
C3—H3	0.9500	C13—C14	1.385 (4)
C4—C5	1.417 (3)	C13—H13	0.9500
C4—C9	1.509 (3)	C14—C15	1.375 (5)
C5—C6	1.388 (3)	C14—H14	0.9500
C5—C10	1.504 (3)	C15—C16	1.384 (4)
C6—C7	1.376 (3)	C16—C17	1.380 (3)
C6—H6	0.9500	C16—H16	0.9500
C8—C11	1.486 (3)	C17—H17	0.9500
			
O2—S1—C1	108.38 (11)	H9A—C9—H9C	109.5
O2—S1—C12	106.81 (11)	H9B—C9—H9C	109.5
C1—S1—C12	97.93 (11)	C5—C10—H10A	109.5
C8—O1—C7	106.33 (16)	C5—C10—H10B	109.5
C8—C1—C2	107.05 (19)	H10A—C10—H10B	109.5
C8—C1—S1	122.76 (16)	C5—C10—H10C	109.5
C2—C1—S1	129.82 (16)	H10A—C10—H10C	109.5
C7—C2—C3	118.5 (2)	H10B—C10—H10C	109.5
C7—C2—C1	104.66 (18)	C8—C11—H11A	109.5
C3—C2—C1	136.8 (2)	C8—C11—H11B	109.5
C4—C3—C2	119.4 (2)	H11A—C11—H11B	109.5
C4—C3—H3	120.3	C8—C11—H11C	109.5
C2—C3—H3	120.3	H11A—C11—H11C	109.5
C3—C4—C5	120.54 (18)	H11B—C11—H11C	109.5
C3—C4—C9	119.5 (2)	C13—C12—C17	121.3 (2)
C5—C4—C9	120.0 (2)	C13—C12—S1	118.0 (2)
C6—C5—C4	120.2 (2)	C17—C12—S1	120.38 (17)
C6—C5—C10	119.0 (2)	C12—C13—C14	119.0 (3)
C4—C5—C10	120.84 (19)	C12—C13—H13	120.5
C7—C6—C5	117.7 (2)	C14—C13—H13	120.5
C7—C6—H6	121.1	C15—C14—C13	119.5 (2)
C5—C6—H6	121.1	C15—C14—H14	120.3
C6—C7—O1	125.39 (19)	C13—C14—H14	120.3
C6—C7—C2	123.72 (19)	C14—C15—C16	121.7 (2)
O1—C7—C2	110.88 (19)	C14—C15—Br1	119.5 (2)
C1—C8—O1	111.08 (18)	C16—C15—Br1	118.8 (2)
C1—C8—C11	133.3 (2)	C17—C16—C15	118.7 (3)
O1—C8—C11	115.63 (19)	C17—C16—H16	120.7
C4—C9—H9A	109.5	C15—C16—H16	120.7
C4—C9—H9B	109.5	C16—C17—C12	119.8 (2)
H9A—C9—H9B	109.5	C16—C17—H17	120.1
C4—C9—H9C	109.5	C12—C17—H17	120.1
			
O2—S1—C1—C8	115.6 (2)	C1—C2—C7—C6	179.3 (2)
C12—S1—C1—C8	−133.6 (2)	C3—C2—C7—O1	−179.8 (2)
O2—S1—C1—C2	−56.5 (3)	C1—C2—C7—O1	−0.2 (3)
C12—S1—C1—C2	54.3 (2)	C2—C1—C8—O1	−0.3 (3)
C8—C1—C2—C7	0.3 (3)	S1—C1—C8—O1	−174.01 (17)
S1—C1—C2—C7	173.37 (19)	C2—C1—C8—C11	179.6 (3)
C8—C1—C2—C3	179.8 (3)	S1—C1—C8—C11	6.0 (4)
S1—C1—C2—C3	−7.1 (4)	C7—O1—C8—C1	0.2 (3)
C7—C2—C3—C4	−0.2 (3)	C7—O1—C8—C11	−179.7 (2)
C1—C2—C3—C4	−179.7 (3)	O2—S1—C12—C13	−15.3 (2)
C2—C3—C4—C5	0.1 (4)	C1—S1—C12—C13	−127.3 (2)
C2—C3—C4—C9	−179.7 (2)	O2—S1—C12—C17	171.18 (18)
C3—C4—C5—C6	0.4 (4)	C1—S1—C12—C17	59.2 (2)
C9—C4—C5—C6	−179.8 (2)	C17—C12—C13—C14	0.1 (4)
C3—C4—C5—C10	−179.2 (2)	S1—C12—C13—C14	−173.3 (2)
C9—C4—C5—C10	0.6 (3)	C12—C13—C14—C15	1.2 (4)
C4—C5—C6—C7	−0.9 (3)	C13—C14—C15—C16	−2.2 (4)
C10—C5—C6—C7	178.7 (2)	C13—C14—C15—Br1	176.7 (2)
C5—C6—C7—O1	−179.7 (2)	C14—C15—C16—C17	1.7 (4)
C5—C6—C7—C2	0.8 (4)	Br1—C15—C16—C17	−177.17 (18)
C8—O1—C7—C6	−179.5 (2)	C15—C16—C17—C12	−0.4 (4)
C8—O1—C7—C2	0.0 (3)	C13—C12—C17—C16	−0.5 (4)
C3—C2—C7—C6	−0.3 (4)	S1—C12—C17—C16	172.77 (19)

### Hydrogen-bond geometry (Å, º)

Cg1 and Cg2 are the centroids of the C1/C2/C7/O1/C8 furan ring and the C2–C7 benzene ring, respectively.

**Table:**

*D*—H···*A*	*D*—H	H···*A*	*D*···*A*	*D*—H···*A*
C9—H9*C*···*Cg*1^i^	0.98	2.89	3.537 (4)	124
C11—H11*C*···*Cg*2^ii^	0.98	2.78	3.714 (4)	159

Symmetry codes: (i) *x*, −*y*+1/2, *z*−1/2; (ii) *x*, −*y*+3/2, *z*+1/2.
